# The Budapest Congress

**Published:** 1909-08

**Authors:** 


A Monthly Review of Medicine and Surgery.

EDITOR

WILLIAM WARREN POTTER, M. D.

All communications, whether of a literary or business nature, books for review and exchanges, should be addressed to the editor, 238 DELAWARE AVENUE, BUFFALO, N.Y.

VOL. LXV. AUGUST, 1909. No. 1

### The Budapest Congress

THE Sixteenth International Medical Congress is to be held at Budapest, August 29 to September 4, 1909. It is fortunate, most fortunate, that Budapest was chosen as the place of this important medical gathering. It is the most beautiful city of Central Europe, containing eight hundred thousand persons—twice the size of Buffalo, for example,—a parliament city, the seat of the Austrian government, where also the courts are held and withal, where royalty is seen.

Budapest, though the product of but a few decades in its modern dress and glory, yet dates back to the year 1200, that is, Pest boasts its life from that period, while Buda was the Ofen of the Romans. The twin cities lie astride the Danube, being connected by bridge. As an example of the progressiveness of this wonderful city, it is interesting to learn that here was built the first underground trolley line, the first subway as we say to-day, and which stands as a model in construction and service.

In *Harper's Monthly* for June is an article on "The Danube" by Marie Van Vorst, in which she says "in Budapest one hears "the Czgane music in all its wierd charm, its rude original char-"acter. Its passion and fire kindle the heart of the poorest *aföld* "peasant. The fingers of the skilful Hungarian musician call "forth this enchantment from the most primitive instruments, "and before a tiny fruit-shop in the poor quarter of Budapest one "may see, in his white cloth dress, a rude laborer from the *aföld,* "and watch him touch with the knowledge of a connoisseur one "after another of the primitive fiddles there for sale, drawing "from their strings a rhapsody of sound as sweet as the notes "of the birds upon the Carpathian upland, or as alluring as the "waves of the Danube as it washes his river farm."

We cannot refrain from presenting this beautiful tribute which Mrs. Van Vorst pays to Hungarian music and to the peasant musicians of that remarkable country. Continuing she says: "the restaurants and cafés in Budapest are famous for "their music; but the most perfect voice of the steppes is to be "heard in the Hungarian villages themselves. At the table of "some rich peasant, as the wine fills the glass and the housewife "prepares the delectable *golyas*, the native musicians gather, in "the gay dress whose fashion has not changed for five centuries. "These dark faces are aflame with ecstasy, and their art is wild "and original. They seem to bring back the past itself, and to "keep prisoner for a time the magic of the wild old days. They "seem lords again.—kings of the plains, kings of the river, coun- "try,—and the veins of the desolate peaks light up, watch-fires "burn on the hillsides, and Hungarians and Magyars of ancient "time file by through the low farm room as the musicians play."

And she adds, "There is no prouder nobility than the Hun- "garian, and entry into the society of Austria and Hungary is "the most difficult in Europe."

We have quoted from Mrs. Van Vorst's article somewhat at length because, first, it is most interesting; and, second, because it is beautifully written in charmingly graceful rhetoric; and, third because Budapest is the City of the Congress this year. We add Dr. Charles Wood Fassett's notice as it gives valuable information to those interested in the Congress.

For the benefit of those who contemplate attending this congress, we would state that ample arrangements have been made for hotel accommodations in Budapest, a large number of rooms having been engaged a year or more ago, in the Hotel Hungaria, for the members of the American party. Reservation should be made at once, to insure good rooms. Those who join this party will have no worry as to details, a competent guide being in constant attendance. The cost of the entire trip, including a week's board in Budapest, meals en route, railroad fare, tips, first-class steamship both ways, carriages for sight-seeing, visiting hospitals; forty-one days, $395.00, sailing from New York, August 12. Full information and itinerary may be obtained by addressing Dr. Charles Wood Fassett, St. Joseph, Mo. (New York Headquarters, Grand Hotel, Broadway and 31st street; Atlantic City address, Grand Atlantic Hotel).

Those who desire membership in the congress may send their application to Dr. J. H. Musser, chairman American Committee, Philadelphia, accompanied by a fee of $5.00, and professional card.

---

JUSTICE Erlanger, in a decision rendered July 16, 1909, said he would not care to assume the responsibility of permitting the release of Mary Mallon from North Brother Island, where she has been virtually a prisoner for over two years, being known as

"Typhoid Mary." Miss Mallon had brought habeas corpus proceedings to obtain her release. The Court said that on further examination she may renew her application, or ask for the appointment of a referee to take testimony.

Although Miss Mallon has never suffered from typhoid fever the health authorities say that she carries thousands of the germs of the disease, and that she would be a menace to public health if permitted to go at large. She was once cook in the household of J. Coleman Drayton, and it was stated that here as well as in other places where she worked she left a trail of typhoid fever victims.

She was sent to North Brother Island in March, 1907, where she has since been an isolated prisoner. In her application for freedom the unfortunate woman denies that she has typhoid, or was ever responsible for the spread of the disease.

Dr. Westmoreland, of the Riverside Hospital, on North Brother Island, said Mary Mallon was placed in the hospital at the instance of the Department of Health, and he had examined her and found she was infected with typhoid germs. Dr. George A. Soper, of the Health Department, also made an affidavit, in which he stated that he investigated the case and its history and found that during the period of eight years immediately prior to her confinement in the hospital, she had been employed in eight different families, and that in seven of them typhoid fever broke out a few weeks after her appearance. No less than twenty-six cases of typhoid fever could be traced to her before she was taken to the hospital.

Justice Erlanger in his decision expresses sympathy with Miss Mallon, but says that the court must protect the community.

---

Believing that New York ought to follow the example of many other cities in a crusade against the housefly, the committee on pollution of New York waters, representing the Merchants' Association, has begun an active warfare against this household pest. Several thousand circulars, giving information as to the dangers lurking in the prevalence of flies, are ready for circulation, and a campaign of reaching the people by means of advertisements has been instituted. The committee in charge includes Dr. Albert Vander Veer, J. Pierpont Morgan, John Y. Culyer, Daniel D. Jackson, and Edward Hatch, Jr., chairman.

---

THE *Lancet,* the great English medical periodical, says that Englishmen and Americans eat too much. It especially advises people of middle age and older to practise self-restraint in the matter of food. "As the fire of life burns less fiercely and the output of energy is smaller," says the writer, "so the fuel supplied should be reduced that the system may not be clogged with ashes and half-burnt cinders, whereby the activity of the whole machine is from time to time impaired and may even be prematurely arrested."

---

MEDICAL assistance by wireless is the latest triumph added to the list of achievements in this branch of telegraphy, according to the wireless operator on the steamship Cartago, which arrived in New Orleans, June 27, 1909.

While the vessel was one hundred and fifty miles off Swan Island a wireless message addressed to the surgeon of the Cartago was picked up. It read: "Employe John D. Graham, Swan Island, suffering blood poison result of cutting foot. Send prescription." •The surgeon of the Cartoga immediately sent the prescription by wireless. Later messages told of the injured man's gradual improvement.

---

IN a letter to *"The Journal of the American Medical Association,"* Professor W. W. Keen, of Philadelphia, makes the following statement: A few weeks ago he noticed in the pages of an American periodical what purported to be a quotation from an address by the distinguished British surgeon Sir Frederic Treves from which the unsuspecting reader would get the impression that Sir Frederick was opposed to vivisection. A marked copy of the article having been sent to him, however, he indignantly repudiated the sentiments imputed to him, saying in a letter to Professor Keen. "I notice that the methods which have "covered the antivivisection party in this country (England) "with contempt and ridicule are being reproduced in America. " . . . I am, as every sane medical man must be, an ardent "supporter of vivisection." The incident deserves notice for two reasons. Sir Frederick Treves is one of the foremost surgeons in the world. It was he who performed the operation on King Edward for appendicitis seven years ago, and he was surgeon-general of the British forces in South Africa in 1900. Further evidence of his standing, not as a leader of his profession, but as a representative of the cause of humanity, may be found in the fact that he is chairman of the executive committee of the British Red Cross. It is particularly important that the opinions of such a man should not be misrepresented.—*Tribune,* 6, 30, '09.

---

DR. JOHN H. PRYOR has been appointed by President Thomas B. Lockwood of the Buffalo Association for the Relief and Control of Tuberculosis as physician in charge of the Tuberculosis Dispensary at 165 Swan street. The committee acting with Dr. Pryor will be Frederick Almy, Irving S. Underhill and John R. Shillady.

The new committee for social work consists of John R. Shillady, Dr. James Otto, Miss Marie Love, Miss Mary A. Lewis and Mrs. Anna B. Fox.

---

The throbbing and vibration of the engines of a modern steamer, according to the *Popular Magazine,* have a most extraordinary effect upon the human heart. Let it be said at once that ocean traveling does not in any way injure the heart; on the contrary, it benefits it, as it does the general health. But the vibration of the machinery is transmitted to this vital organ with the most extraordinary results so far as medical examination is concerned. A ship's doctor will tell you that when he listens through his stethoscope to the beating of a man's heart at sea, it seems as though at every moment the heart would stop.

With sturdy and invalid passengers the case is the same. The heart appears to the doctor as though every beat would be its last. This being the case, it is exceedingly difficult for the physician to ascertain the true condition of the traveler's health, and he generally resorts to the expedient of slinging his patient in a hammock, where the vibration is considerably lessened, though no device can overcome it altogether.

